
JOURNAL INFORMATION
==============================
NLM Title Abbreviation: ScientificWorldJournal
Journal ID: ScientificWorldJournal
NLM Journal ID: 101131163
Journal ID: TSWJ

The Scientific World Journal

ISSN: 2356-6140
EISSN: 1537-744X
Publisher: Wiley

ARTICLE INFORMATION
==============================
PMCID: PMC6009721
DOI: 10.1100/tsw.2002.807
Article ID: 687465
Article version: 1
Subjects: Book Review

Book Review—Immunology of Infectious Diseases

Taylor-Robinson Andrew *
School of Biology, University of Leeds, UK
*Andrew Taylor-Robinson: bgyawtr@leeds.ac.uk
Publication date: 2002
Electronic publication date: 2002 Jun 11
Volume: 2
First page: 1594
Last page: 1596
Received 2002 Apr 24; Accepted 2002 Apr 24
Copyright: Copyright © 2002 Andrew Taylor-Robinson.
Copyright year: 2002
License: This is an open access article distributed under the Creative Commons Attribution License, which permits unrestricted use, distribution, and reproduction in any medium, provided the original work is properly cited.
License URL: https://creativecommons.org/licenses/by/3.0/

==============================
**Book Review**
*TheScientificWorldJOURNAL* (2002) 2, 1594–1596
ISSN 1537-744X; DOI 10.1100/tsw.2002.807

TheScientificWorld
www.thescientificworld.com

### Book Review — *Immunology of Infectious Diseases*

Andrew Taylor-Robinson
*School of Biology, University of Leeds, U.K.*

E-mail: bgyawtr@leeds.ac.uk

*Received April 24, 2002; Accepted April 24, 2002; Published June 11, 2002*

---

**DOMAINS:** immunology, applied microbiology, microbiology, microbiology (parasitology), microbiology (bacteriology), virology, cell biology, infection, pathology, microbial pathogenesis, vaccine discovery

---

### Immunology of Infectious Diseases

Edited by **Stephan H.E. Kaufmann** (Max Planck Institute for Infection Biology, Berlin, Germany), **Alan Sher** (National Institute of Allergy and Infectious Diseases, Bethesda, U.S.) and **Rafi Ahmed** (Emory University School of Medicine, Atlanta, U.S.)

American Society for Microbiology (ASM) Press, Washington DC, 2002

$115.95 hardback (xi + 495 pages) ISBN 1-55581-214-7

The editors of *Immunology of Infectious Diseases* state that their aim is to provide a textbook to fill the gap that has emerged since the study of microbiology and the science of immunology diverged from a common origin over the last 2 decades, a time that has witnessed significant progress in our understanding of the mechanisms of immunity to infectious diseases. To this end, they have assembled a team of internationally acknowledged researchers to write on their respective areas of expertise. The book is a large, single volume approaching 500 pages in length which is divided into eight sections considering pathogens, the anti-infective immune response, innate and then acquired immunity, before turning to pathology, evasion and latency, immunogenetics, and finally immune intervention. These are all key areas and, read as a whole, the 31 chapters provide a valuable synthesis of the current state of infectious disease immunology and through this afford an insight into how the immune and inflammatory systems are associated and regulated.

The book starts with a well-written introductory overview of the aetiological agents of disease, namely bacterial, viral, fungal, and parasitic pathogens. Bacteria and parasites are considered by focussing on specific examples in which the authors are specialists whereas viruses

©2002 with author.

and fungi are treated more in terms of general principles such as tissue tropism, genetic instability, and subversion of the immune response. There then follows the meat of the book, sections on innate and acquired immunity, including excellent chapters on phagocytes, defensins and cathelicidins, immunological memory, and regional immune responses. The section on pathology is arguably the most interesting for the unbiased reader because it brings home the fact that virtually all insults to an individual, be they acute or chronic, induce cytokine responses and that nearly every disease has a component that involves the function of cytokines. Application of this knowledge is contributing immensely to the growing impact of molecular medicine in providing new approaches to treatment of infectious diseases. In my opinion, however, an omission, here and elsewhere, is discussion of cytokine-induced modulation of infection and potentiation of vaccine efficacy by cytokine adjuvants.

One of the general strengths of the book is that research in species other than humans is not only documented but considered in terms of its importance to our understanding of human disease. The exception to this is that there are two chapters that deal with the immunogenetics of the host response to bacteria, one of which is devoted to studies in man and the other to studies in mice. This lack of integration serves only to highlight the value of uniting such research with the ultimate aim of successfully manipulating the immune response to defend against the deleterious effects of infection. Such steps that have been taken towards immune therapy and vaccination are described in relation to tuberculosis and AIDS.

While Kaufmann, Sher, and Ahmed should be congratulated for compiling this comprehensive compendium, their editorial touch has been light. This was sufficient to ensure that all authors followed the brief to discuss the ‘dialogue’ between pathogens and the host immune system, but did not extend to cross-referencing between sections to provide common links. Hence, each chapter is free-standing and does not relate to any other. One consequence of this is that some common aspects are discussed repeatedly throughout the book. The dichotomy between T helper 1 and 2 lymphocytes is considered in several chapters, albeit from slightly different perspectives. Likewise, the mechanism of evasion of the host immune system is a recurrent theme. In addition, basic facts and figures of each disease crop up repeatedly. For example, it is not necessary to find out on four separate occasions that tuberculosis infects a third of the world’s population, killing 3 million people each year. Another way in which this book could be improved is by a much greater use of figures and tables, which are employed effectively in some but not all chapters. Colour plates feature in the centre of the book in a separate section but the technology exists for these to be inserted adjacent to the relevant text. A pleasing feature is that all chapters end with a sizeable list of original cited references as well as handfull of lead-in reviews of more detailed coverage (as recent as the year 2000).

This book is of course no different to all other biomedical texts in being a little out of date by the time it is published. This is an accepted frustration of the prolonged gestation period that besets multi-authored volumes. Hence, for instance, there is no mention of dendritic cell heterogeneity and its functional significance to T cell priming that has come to light recently. As this serves to indicate how rapidly moving a field infectious disease immunology is, perhaps this and other advances will be covered in a revised edition in the not too distant future?

To recommend as a set text for teaching undergraduates weaned on a time-honoured diet of Roitt, Brostoff, and Male’s *Immunology* or Janeway and Travers’ *Immunobiology*, this book may be slightly too advanced. It should, however, find its way onto a more specialised list for recommended reading and thereby merit a place on the shelf of your departmental or institutional library. For Masters level upwards, it provides a valuable resource that places the role of each researcher’s favourite cell or cytokine into a wider, networked, context and explains clearly the functions and interplay of the innate and adaptive immune responses to any given pathogen.

**This article should be referenced as follows:**

Taylor-Robinson, A. (2002) Book review – *Immunology of Infectious Diseases*. *TheScientificWorldJOURNAL* **2**, 1594–1596.
